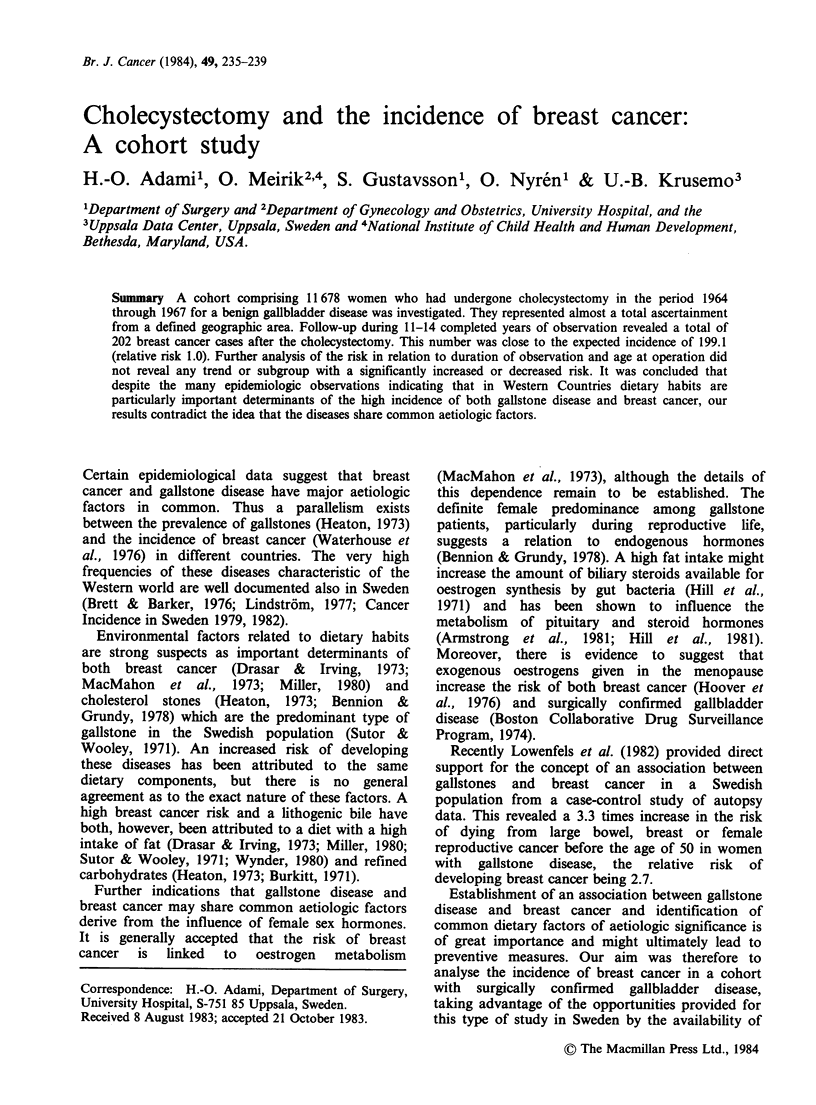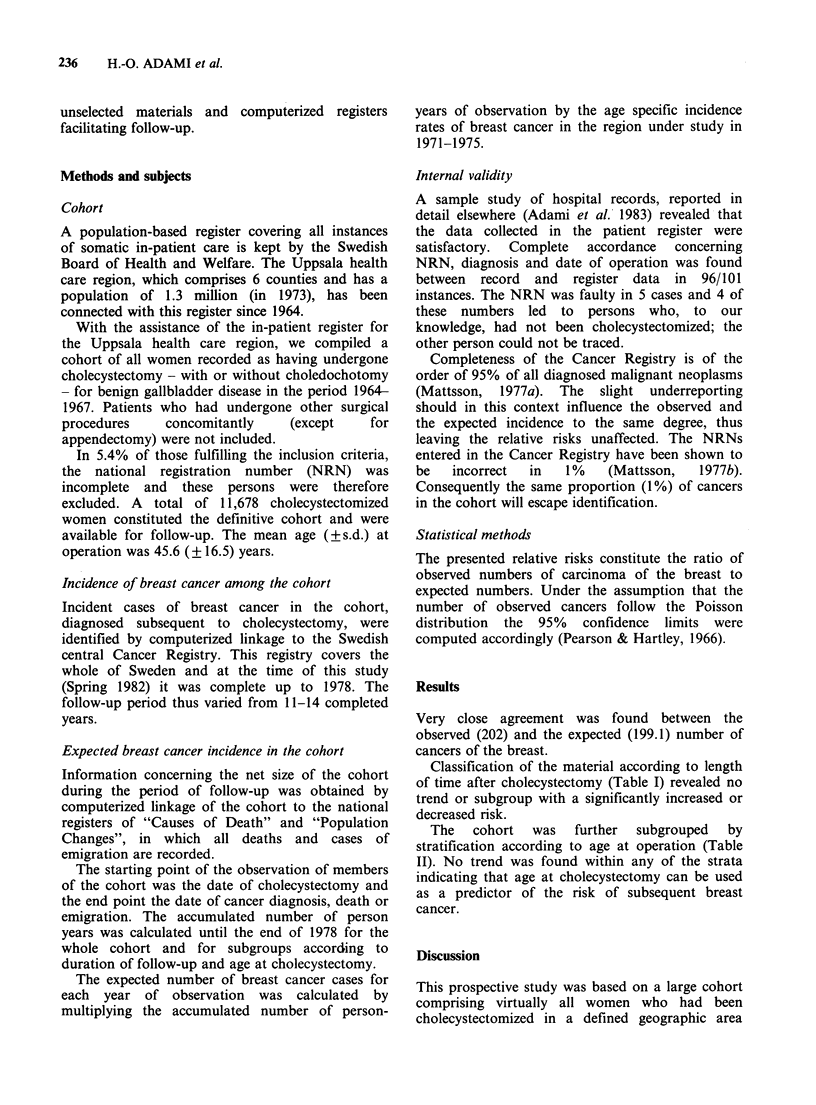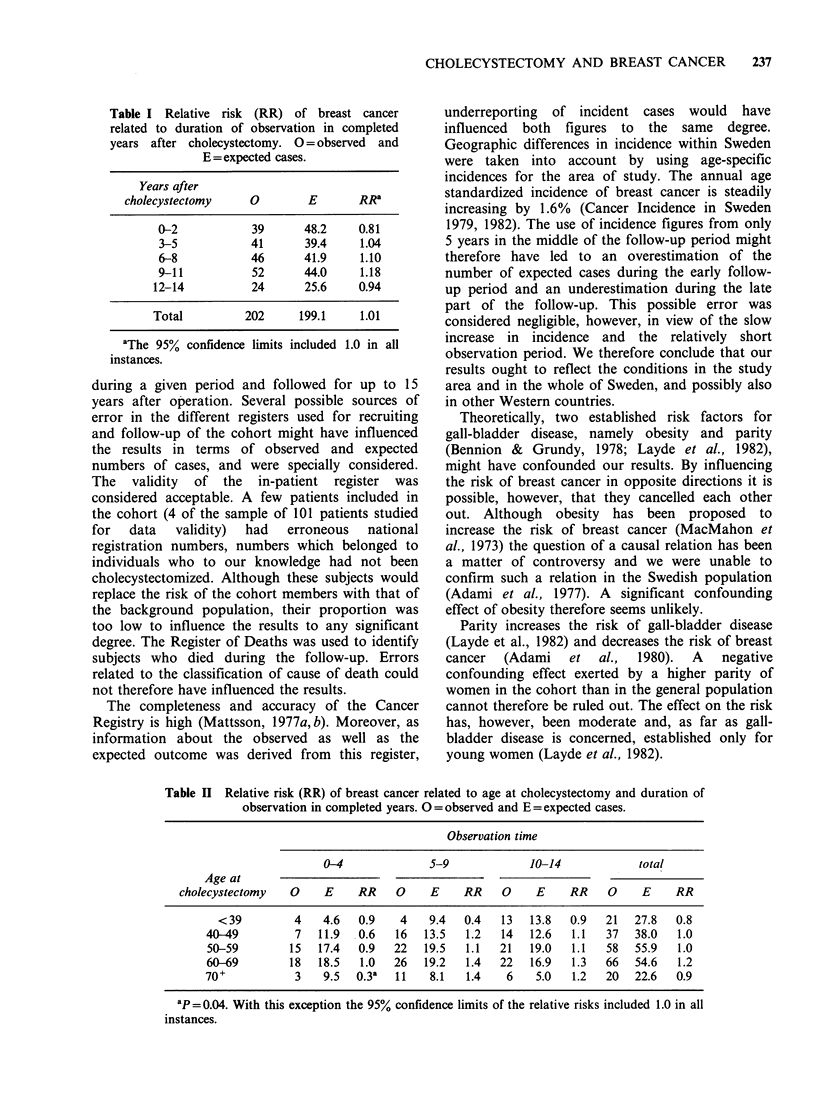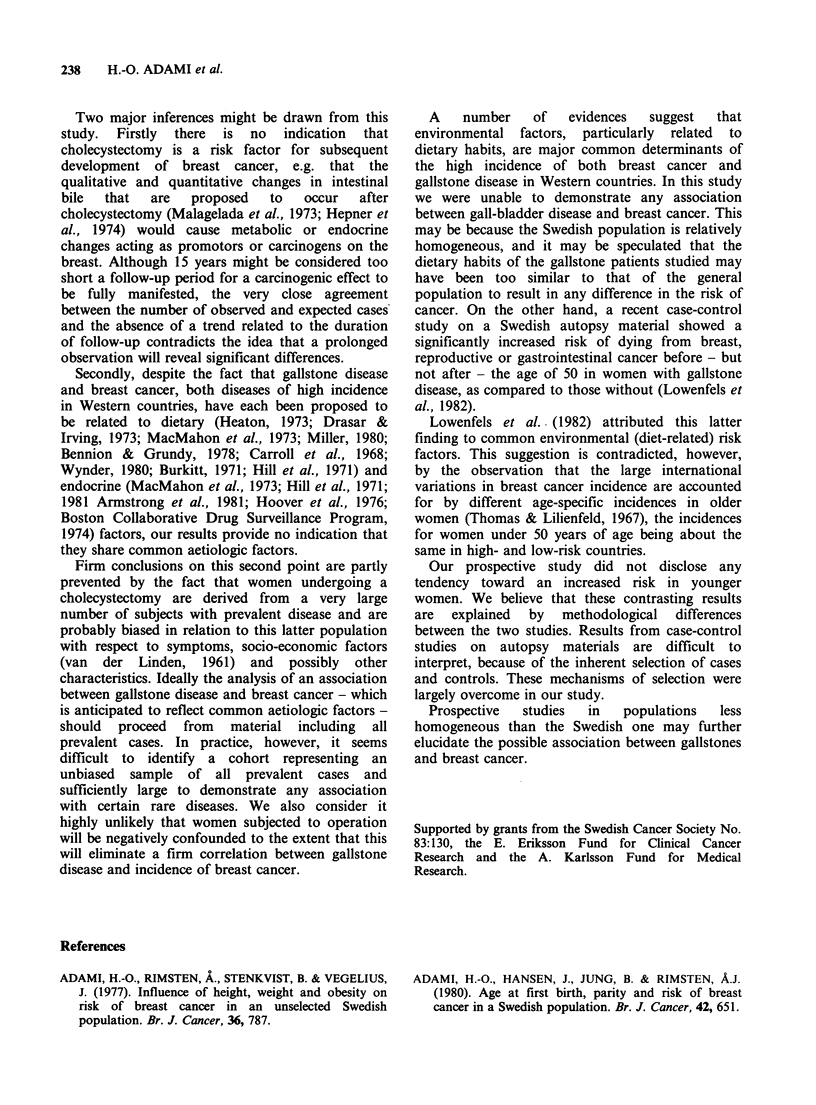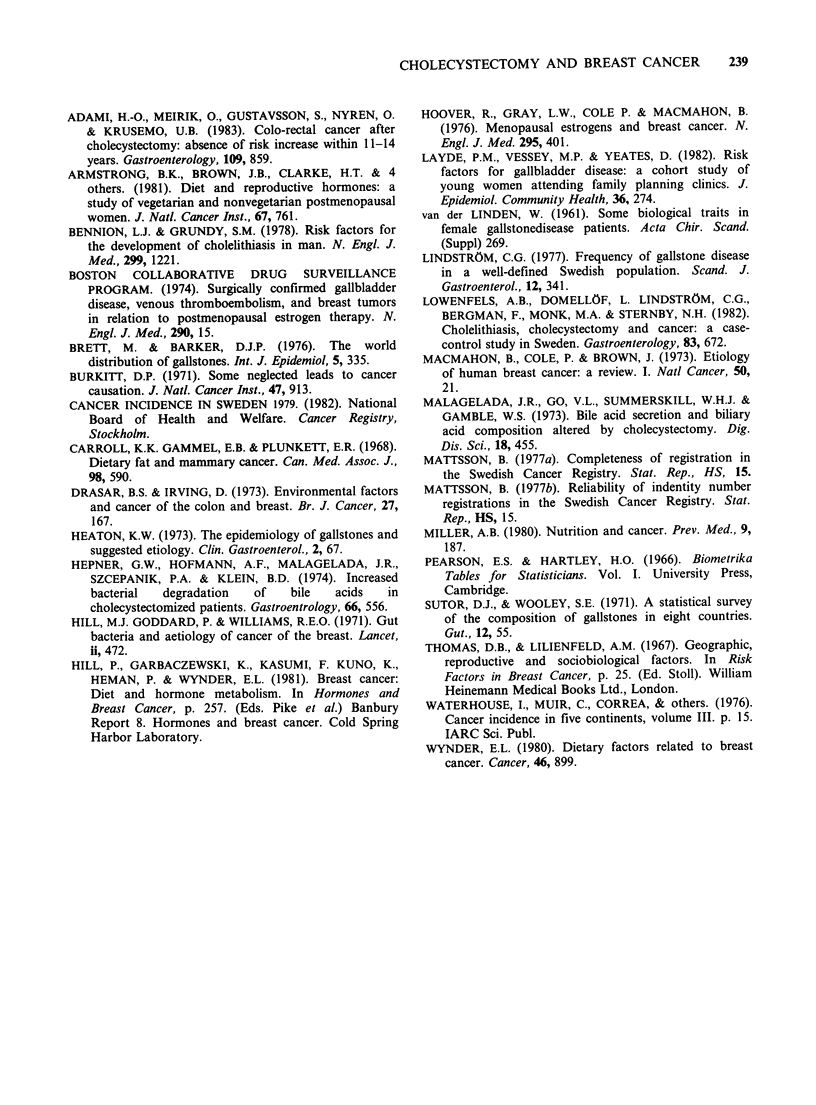# Cholecystectomy and the incidence of breast cancer: a cohort study.

**DOI:** 10.1038/bjc.1984.36

**Published:** 1984-02

**Authors:** H. O. Adami, O. Meirik, S. Gustavsson, O. Nyrén, U. B. Krusemo

## Abstract

A cohort comprising 11678 women who had undergone cholecystectomy in the period 1964 through 1967 for a benign gallbladder disease was investigated. They represented almost a total ascertainment from a defined geographic area. Follow-up during 11-14 completed years of observation revealed a total of 202 breast cancer cases after the cholecystectomy. This number was close to the expected incidence of 199.1 (relative risk 1.0). Further analysis of the risk in relation to duration of observation and age at operation did not reveal any trend or subgroup with a significantly increased or decreased risk. It was concluded that despite the many epidemiologic observations indicating that in Western Countries dietary habits are particularly important determinants of the high incidence of both gallstone disease and breast cancer, our results contradict the idea that the diseases share common aetiologic factors.


*Br. J. Cancer* (1984), **49**, 235–239

### Cholecystectomy and the incidence of breast cancer: A cohort study

H.-O. Adami[1], O. Meirik[2,4], S. Gustavsson[1], O. Nyrén[1] & U.-B. Krusemo[3]

[1]*Department of Surgery and* [2]*Department of Gynecology and Obstetrics, University Hospital, and the* [3]*Uppsala Data Center, Uppsala, Sweden and* [4]*National Institute of Child Health and Human Development, Bethesda, Maryland, USA.*

**Summary** A cohort comprising 11 678 women who had undergone cholecystectomy in the period 1964 through 1967 for a benign gallbladder disease was investigated. They represented almost a total ascertainment from a defined geographic area. Follow-up during 11–14 completed years of observation revealed a total of 202 breast cancer cases after the cholecystectomy. This number was close to the expected incidence of 199.1 (relative risk 1.0). Further analysis of the risk in relation to duration of observation and age at operation did not reveal any trend or subgroup with a significantly increased or decreased risk. It was concluded that despite the many epidemiologic observations indicating that in Western Countries dietary habits are particularly important determinants of the high incidence of both gallstone disease and breast cancer, our results contradict the idea that the diseases share common aetiologic factors.

Certain epidemiological data suggest that breast cancer and gallstone disease have major aetiologic factors in common. Thus a parallelism exists between the prevalence of gallstones (Heaton, 1973) and the incidence of breast cancer (Waterhouse *et al.*, 1976) in different countries. The very high frequencies of these diseases characteristic of the Western world are well documented also in Sweden (Brett & Barker, 1976; Lindström, 1977; Cancer Incidence in Sweden 1979, 1982).

Environmental factors related to dietary habits are strong suspects as important determinants of both breast cancer (Drasar & Irving, 1973; MacMahon *et al.*, 1973; Miller, 1980) and cholesterol stones (Heaton, 1973; Bennion & Grundy, 1978) which are the predominant type of gallstone in the Swedish population (Sutor & Wooley, 1971). An increased risk of developing these diseases has been attributed to the same dietary components, but there is no general agreement as to the exact nature of these factors. A high breast cancer risk and a lithogenic bile have both, however, been attributed to a diet with a high intake of fat (Drasar & Irving, 1973; Miller, 1980; Sutor & Wooley, 1971; Wynder, 1980) and refined carbohydrates (Heaton, 1973; Burkitt, 1971).

Further indications that gallstone disease and breast cancer may share common aetiologic factors derive from the influence of female sex hormones. It is generally accepted that the risk of breast cancer is linked to oestrogen metabolism (MacMahon *et al.*, 1973), although the details of this dependence remain to be established. The definite female predominance among gallstone patients, particularly during reproductive life, suggests a relation to endogenous hormones (Bennion & Grundy, 1978). A high fat intake might increase the amount of biliary steroids available for oestrogen synthesis by gut bacteria (Hill *et al.*, 1971) and has been shown to influence the metabolism of pituitary and steroid hormones (Armstrong *et al.*, 1981; Hill *et al.*, 1981). Moreover, there is evidence to suggest that exogenous oestrogens given in the menopause increase the risk of both breast cancer (Hoover *et al.*, 1976) and surgically confirmed gallbladder disease (Boston Collaborative Drug Surveillance Program, 1974).

Recently Lowenfels *et al.* (1982) provided direct support for the concept of an association between gallstones and breast cancer in a Swedish population from a case-control study of autopsy data. This revealed a 3.3 times increase in the risk of dying from large bowel, breast or female reproductive cancer before the age of 50 in women with gallstone disease, the relative risk of developing breast cancer being 2.7.

Establishment of an association between gallstone disease and breast cancer and identification of common dietary factors of aetiologic significance is of great importance and might ultimately lead to preventive measures. Our aim was therefore to analyse the incidence of breast cancer in a cohort with surgically confirmed gallbladder disease, taking advantage of the opportunities provided for this type of study in Sweden by the availability of

Correspondence: H.-O. Adami, Department of Surgery, University Hospital, S-751 85 Uppsala, Sweden.
Received 8 August 1983; accepted 21 October 1983.

© The Macmillan Press Ltd., 1984

unselected materials and computerized registers facilitating follow-up.

### Methods and subjects

### *Cohort*

A population-based register covering all instances of somatic in-patient care is kept by the Swedish Board of Health and Welfare. The Uppsala health care region, which comprises 6 counties and has a population of 1.3 million (in 1973), has been connected with this register since 1964.

With the assistance of the in-patient register for the Uppsala health care region, we compiled a cohort of all women recorded as having undergone cholecystectomy – with or without choledochotomy – for benign gallbladder disease in the period 1964–1967. Patients who had undergone other surgical procedures concomitantly (except for appendectomy) were not included.

In 5.4% of those fulfilling the inclusion criteria, the national registration number (NRN) was incomplete and these persons were therefore excluded. A total of 11,678 cholecystectomized women constituted the definitive cohort and were available for follow-up. The mean age ($\pm$s.d.) at operation was 45.6 ($\pm$16.5) years.

### *Incidence of breast cancer among the cohort*

Incident cases of breast cancer in the cohort, diagnosed subsequent to cholecystectomy, were identified by computerized linkage to the Swedish central Cancer Registry. This registry covers the whole of Sweden and at the time of this study (Spring 1982) it was complete up to 1978. The follow-up period thus varied from 11–14 completed years.

### *Expected breast cancer incidence in the cohort*

Information concerning the net size of the cohort during the period of follow-up was obtained by computerized linkage of the cohort to the national registers of "Causes of Death" and "Population Changes", in which all deaths and cases of emigration are recorded.

The starting point of the observation of members of the cohort was the date of cholecystectomy and the end point the date of cancer diagnosis, death or emigration. The accumulated number of person years was calculated until the end of 1978 for the whole cohort and for subgroups according to duration of follow-up and age at cholecystectomy.

The expected number of breast cancer cases for each year of observation was calculated by multiplying the accumulated number of person-years of observation by the age specific incidence rates of breast cancer in the region under study in 1971–1975.

### *Internal validity*

A sample study of hospital records, reported in detail elsewhere (Adami *et al.* 1983) revealed that the data collected in the patient register were satisfactory. Complete accordance concerning NRN, diagnosis and date of operation was found between record and register data in 96/101 instances. The NRN was faulty in 5 cases and 4 of these numbers led to persons who, to our knowledge, had not been cholecystectomized; the other person could not be traced.

Completeness of the Cancer Registry is of the order of 95% of all diagnosed malignant neoplasms (Mattsson, 1977*a*). The slight underreporting should in this context influence the observed and the expected incidence to the same degree, thus leaving the relative risks unaffected. The NRNs entered in the Cancer Registry have been shown to be incorrect in 1% (Mattsson, 1977*b*). Consequently the same proportion (1%) of cancers in the cohort will escape identification.

### *Statistical methods*

The presented relative risks constitute the ratio of observed numbers of carcinoma of the breast to expected numbers. Under the assumption that the number of observed cancers follow the Poisson distribution the 95% confidence limits were computed accordingly (Pearson & Hartley, 1966).

### Results

Very close agreement was found between the observed (202) and the expected (199.1) number of cancers of the breast.

Classification of the material according to length of time after cholecystectomy (Table I) revealed no trend or subgroup with a significantly increased or decreased risk.

The cohort was further subgrouped by stratification according to age at operation (Table II). No trend was found within any of the strata indicating that age at cholecystectomy can be used as a predictor of the risk of subsequent breast cancer.

### Discussion

This prospective study was based on a large cohort comprising virtually all women who had been cholecystectomized in a defined geographic area

**Table I** Relative risk (RR) of breast cancer related to duration of observation in completed years after cholecystectomy. O = observed and E = expected cases.

| *Years after cholecystectomy* | *O* | *E* | *RR*[a] |
|---|---|---|---|
| 0–2 | 39 | 48.2 | 0.81 |
| 3–5 | 41 | 39.4 | 1.04 |
| 6–8 | 46 | 41.9 | 1.10 |
| 9–11 | 52 | 44.0 | 1.18 |
| 12–14 | 24 | 25.6 | 0.94 |
| Total | 202 | 199.1 | 1.01 |

[a]The 95% confidence limits included 1.0 in all instances.

during a given period and followed for up to 15 years after operation. Several possible sources of error in the different registers used for recruiting and follow-up of the cohort might have influenced the results in terms of observed and expected numbers of cases, and were specially considered. The validity of the in-patient register was considered acceptable. A few patients included in the cohort (4 of the sample of 101 patients studied for data validity) had erroneous national registration numbers, numbers which belonged to individuals who to our knowledge had not been cholecystectomized. Although these subjects would replace the risk of the cohort members with that of the background population, their proportion was too low to influence the results to any significant degree. The Register of Deaths was used to identify subjects who died during the follow-up. Errors related to the classification of cause of death could not therefore have influenced the results.

The completeness and accuracy of the Cancer Registry is high (Mattsson, 1977*a*, *b*). Moreover, as information about the observed as well as the expected outcome was derived from this register, underreporting of incident cases would have influenced both figures to the same degree. Geographic differences in incidence within Sweden were taken into account by using age-specific incidences for the area of study. The annual age standardized incidence of breast cancer is steadily increasing by 1.6% (Cancer Incidence in Sweden 1979, 1982). The use of incidence figures from only 5 years in the middle of the follow-up period might therefore have led to an overestimation of the number of expected cases during the early follow-up period and an underestimation during the late part of the follow-up. This possible error was considered negligible, however, in view of the slow increase in incidence and the relatively short observation period. We therefore conclude that our results ought to reflect the conditions in the study area and in the whole of Sweden, and possibly also in other Western countries.

Theoretically, two established risk factors for gall-bladder disease, namely obesity and parity (Bennion & Grundy, 1978; Layde *et al.*, 1982), might have confounded our results. By influencing the risk of breast cancer in opposite directions it is possible, however, that they cancelled each other out. Although obesity has been proposed to increase the risk of breast cancer (MacMahon *et al.*, 1973) the question of a causal relation has been a matter of controversy and we were unable to confirm such a relation in the Swedish population (Adami *et al.*, 1977). A significant confounding effect of obesity therefore seems unlikely.

Parity increases the risk of gall-bladder disease (Layde et al., 1982) and decreases the risk of breast cancer (Adami *et al.*, 1980). A negative confounding effect exerted by a higher parity of women in the cohort than in the general population cannot therefore be ruled out. The effect on the risk has, however, been moderate and, as far as gall-bladder disease is concerned, established only for young women (Layde *et al.*, 1982).

**Table II** Relative risk (RR) of breast cancer related to age at cholecystectomy and duration of observation in completed years. O = observed and E = expected cases.

| | *Observation time* | | | | | | | | | | | |
|---|---|---|---|---|---|---|---|---|---|---|---|---|
| | *0–4* | | | *5–9* | | | *10–14* | | | *total* | | |
| *Age at cholecystectomy* | *O* | *E* | *RR* | *O* | *E* | *RR* | *O* | *E* | *RR* | *O* | *E* | *RR* |
| <39 | 4 | 4.6 | 0.9 | 4 | 9.4 | 0.4 | 13 | 13.8 | 0.9 | 21 | 27.8 | 0.8 |
| 40–49 | 7 | 11.9 | 0.6 | 16 | 13.5 | 1.2 | 14 | 12.6 | 1.1 | 37 | 38.0 | 1.0 |
| 50–59 | 15 | 17.4 | 0.9 | 22 | 19.5 | 1.1 | 21 | 19.0 | 1.1 | 58 | 55.9 | 1.0 |
| 60–69 | 18 | 18.5 | 1.0 | 26 | 19.2 | 1.4 | 22 | 16.9 | 1.3 | 66 | 54.6 | 1.2 |
| $70^+$ | 3 | 9.5 | 0.3[a] | 11 | 8.1 | 1.4 | 6 | 5.0 | 1.2 | 20 | 22.6 | 0.9 |

[a]$P = 0.04$. With this exception the 95% confidence limits of the relative risks included 1.0 in all instances.

Two major inferences might be drawn from this study. Firstly there is no indication that cholecystectomy is a risk factor for subsequent development of breast cancer, e.g. that the qualitative and quantitative changes in intestinal bile that are proposed to occur after cholecystectomy (Malagelada *et al.*, 1973; Hepner *et al.*, 1974) would cause metabolic or endocrine changes acting as promotors or carcinogens on the breast. Although 15 years might be considered too short a follow-up period for a carcinogenic effect to be fully manifested, the very close agreement between the number of observed and expected cases and the absence of a trend related to the duration of follow-up contradicts the idea that a prolonged observation will reveal significant differences.

Secondly, despite the fact that gallstone disease and breast cancer, both diseases of high incidence in Western countries, have each been proposed to be related to dietary (Heaton, 1973; Drasar & Irving, 1973; MacMahon *et al.*, 1973; Miller, 1980; Bennion & Grundy, 1978; Carroll *et al.*, 1968; Wynder, 1980; Burkitt, 1971; Hill *et al.*, 1971) and endocrine (MacMahon *et al.*, 1973; Hill *et al.*, 1971; 1981 Armstrong *et al.*, 1981; Hoover *et al.*, 1976; Boston Collaborative Drug Surveillance Program, 1974) factors, our results provide no indication that they share common aetiologic factors.

Firm conclusions on this second point are partly prevented by the fact that women undergoing a cholecystectomy are derived from a very large number of subjects with prevalent disease and are probably biased in relation to this latter population with respect to symptoms, socio-economic factors (van der Linden, 1961) and possibly other characteristics. Ideally the analysis of an association between gallstone disease and breast cancer – which is anticipated to reflect common aetiologic factors – should proceed from material including all prevalent cases. In practice, however, it seems difficult to identify a cohort representing an unbiased sample of all prevalent cases and sufficiently large to demonstrate any association with certain rare diseases. We also consider it highly unlikely that women subjected to operation will be negatively confounded to the extent that this will eliminate a firm correlation between gallstone disease and incidence of breast cancer.

A number of evidences suggest that environmental factors, particularly related to dietary habits, are major common determinants of the high incidence of both breast cancer and gallstone disease in Western countries. In this study we were unable to demonstrate any association between gall-bladder disease and breast cancer. This may be because the Swedish population is relatively homogeneous, and it may be speculated that the dietary habits of the gallstone patients studied may have been too similar to that of the general population to result in any difference in the risk of cancer. On the other hand, a recent case-control study on a Swedish autopsy material showed a significantly increased risk of dying from breast, reproductive or gastrointestinal cancer before – but not after – the age of 50 in women with gallstone disease, as compared to those without (Lowenfels *et al.*, 1982).

Lowenfels *et al.* (1982) attributed this latter finding to common environmental (diet-related) risk factors. This suggestion is contradicted, however, by the observation that the large international variations in breast cancer incidence are accounted for by different age-specific incidences in older women (Thomas & Lilienfeld, 1967), the incidences for women under 50 years of age being about the same in high- and low-risk countries.

Our prospective study did not disclose any tendency toward an increased risk in younger women. We believe that these contrasting results are explained by methodological differences between the two studies. Results from case-control studies on autopsy materials are difficult to interpret, because of the inherent selection of cases and controls. These mechanisms of selection were largely overcome in our study.

Prospective studies in populations less homogeneous than the Swedish one may further elucidate the possible association between gallstones and breast cancer.

Supported by grants from the Swedish Cancer Society No. 83:130, the E. Eriksson Fund for Clinical Cancer Research and the A. Karlsson Fund for Medical Research.

### References

ADAMI, H.-O., RIMSTEN, Å., STENKVIST, B. & VEGELIUS, J. (1977). Influence of height, weight and obesity on risk of breast cancer in an unselected Swedish population. *Br. J. Cancer*, **36**, 787.

ADAMI, H.-O., HANSEN, J., JUNG, B. & RIMSTEN, Å.J. (1980). Age at first birth, parity and risk of breast cancer in a Swedish population. *Br. J. Cancer*, **42**, 651.

ADAMI, H.-O., MEIRIK, O., GUSTAVSSON, S., NYREN, O. & KRUSEMO, U.B. (1983). Colo-rectal cancer after cholecystectomy: absence of risk increase within 11–14 years. *Gastroenterology*, **109**, 859.

ARMSTRONG, B.K., BROWN, J.B., CLARKE, H.T. & 4 others. (1981). Diet and reproductive hormones: a study of vegetarian and nonvegetarian postmenopausal women. *J. Natl. Cancer Inst.*, **67**, 761.

BENNION, L.J. & GRUNDY, S.M. (1978). Risk factors for the development of cholelithiasis in man. *N. Engl. J. Med.*, **299**, 1221.

BOSTON COLLABORATIVE DRUG SURVEILLANCE PROGRAM. (1974). Surgically confirmed gallbladder disease, venous thromboembolism, and breast tumors in relation to postmenopausal estrogen therapy. *N. Engl. J. Med.*, **290**, 15.

BRETT, M. & BARKER, D.J.P. (1976). The world distribution of gallstones. *Int. J. Epidemiol*, **5**, 335.

BURKITT, D.P. (1971). Some neglected leads to cancer causation. *J. Natl. Cancer Inst.*, **47**, 913.

CANCER INCIDENCE IN SWEDEN 1979. (1982). National Board of Health and Welfare. *Cancer Registry, Stockholm.*

CARROLL, K.K. GAMMEL, E.B. & PLUNKETT, E.R. (1968). Dietary fat and mammary cancer. *Can. Med. Assoc. J.*, **98**, 590.

DRASAR, B.S. & IRVING, D. (1973). Environmental factors and cancer of the colon and breast. *Br. J. Cancer*, **27**, 167.

HEATON, K.W. (1973). The epidemiology of gallstones and suggested etiology. *Clin. Gastroenterol.*, **2**, 67.

HEPNER, G.W., HOFMANN, A.F., MALAGELADA, J.R., SZCEPANIK, P.A. & KLEIN, B.D. (1974). Increased bacterial degradation of bile acids in cholecystectomized patients. *Gastroentrology*, **66**, 556.

HILL, M.J. GODDARD, P. & WILLIAMS, R.E.O. (1971). Gut bacteria and aetiology of cancer of the breast. *Lancet*, **ii**, 472.

HILL, P., GARBACZEWSKI, K., KASUMI, F. KUNO, K., HEMAN, P. & WYNDER, E.L. (1981). Breast cancer: Diet and hormone metabolism. In *Hormones and Breast Cancer*, p. 257. (Eds. Pike *et al.*) Banbury Report 8. Hormones and breast cancer. Cold Spring Harbor Laboratory.

HOOVER, R., GRAY, L.W., COLE P. & MACMAHON, B. (1976). Menopausal estrogens and breast cancer. *N. Engl. J. Med.* **295**, 401.

LAYDE, P.M., VESSEY, M.P. & YEATES, D. (1982). Risk factors for gallbladder disease: a cohort study of young women attending family planning clinics. *J. Epidemiol. Community Health*, **36**, 274.

van der LINDEN, W. (1961). Some biological traits in female gallstonedisease patients. *Acta Chir. Scand.* (Suppl) 269.

LINDSTRÖM, C.G. (1977). Frequency of gallstone disease in a well-defined Swedish population. *Scand. J. Gastroenterol.*, **12**, 341.

LOWENFELS, A.B., DOMELLÖF, L. LINDSTRÖM, C.G., BERGMAN, F., MONK, M.A. & STERNBY, N.H. (1982). Cholelithiasis, cholecystectomy and cancer: a case-control study in Sweden. *Gastroenterology*, **83**, 672.

MACMAHON, B., COLE, P. & BROWN, J. (1973). Etiology of human breast cancer: a review. I. *Natl Cancer*, **50**, 21.

MALAGELADA, J.R., GO, V.L., SUMMERSKILL, W.H.J. & GAMBLE, W.S. (1973). Bile acid secretion and biliary acid composition altered by cholecystectomy. *Dig. Dis. Sci.*, **18**, 455.

MATTSSON, B. (1977*a*). Completeness of registration in the Swedish Cancer Registry. *Stat. Rep., HS,* **15.**

MATTSSON, B. (1977*b*). Reliability of indentity number registrations in the Swedish Cancer Registry. *Stat. Rep.*, **HS**, 15.

MILLER, A.B. (1980). Nutrition and cancer. *Prev. Med.*, **9**, 187.

PEARSON, E.S. & HARTLEY, H.O. (1966). *Biometrika Tables for Statisticians*. Vol. I. University Press, Cambridge.

SUTOR, D.J., & WOOLEY, S.E. (1971). A statistical survey of the composition of gallstones in eight countries. *Gut.*, **12**, 55.

THOMAS, D.B., & LILIENFELD, A.M. (1967). Geographic, reproductive and sociobiological factors. In *Risk Factors in Breast Cancer*, p. 25. (Ed. Stoll). William Heinemann Medical Books Ltd., London.

WATERHOUSE, I., MUIR, C., CORREA, & others. (1976). Cancer incidence in five continents, volume III. p. 15. IARC Sci. Publ.

WYNDER, E.L. (1980). Dietary factors related to breast cancer. *Cancer*, **46**, 899.